# Cardiometabolic Profile, Physical Activity, and Quality of Life in Breast Cancer Survivors after Different Physical Exercise Protocols: A 34-Month Follow-Up Study

**DOI:** 10.3390/jcm12144795

**Published:** 2023-07-20

**Authors:** Valentina Bucciarelli, Francesco Bianco, Andrea Di Blasio, Teresa Morano, Desiree Tuosto, Francesco Mucedola, Serena Di Santo, Alessandra Cimini, Giorgio Napolitano, Ines Bucci, Angela Di Baldassarre, Ettore Cianchetti, Sabina Gallina

**Affiliations:** 1 Cardiovascular Sciences Department—Azienda Ospedaliero-Universitaria delle Marche, 60126 Ancona, Italy; francesco.bianco@ospedaliriuniti.marche.it; 2 Department of Neurosciences, Imaging and Clinical Sciences, “G. d’Annunzio” University, 66100 Chieti, Italy; desiree.tuosto@hotmail.it (D.T.); fra.mucedola@gmail.com (F.M.); sgallina@unich.it (S.G.); 3 Department of Medicine and Aging Sciences, “G. d’Annunzio” University, 66100 Chieti, Italy; andrea.diblasio@unich.it (A.D.B.); moranoteresa@gmail.com (T.M.); serenadisanto@live.it (S.D.S.); giorgio.napolitano@unich.it (G.N.); angela.dibaldassarre@unich.it (A.D.B.); 4 Eusoma Breast Centre, “G. Bernabeo” Hospital, ASL02 Lanciano-Vasto-Chieti, 66026 Ortona, Italy; alessandracimini@hotmail.com (A.C.); ettore.cianchetti@gmail.com (E.C.); 5 Center for Advanced Studies and Technology (CAST), “G. d’Annunzio” University of Chieti-Pescara, 66100 Chieti, Italy; ines.bucci@unich.it

**Keywords:** breast cancer, breast cancer survivors, cancer therapy-related cardiovascular toxicity, hormone therapy, physical exercise, aerobic exercise training, resistance exercise training, cardiometabolic profile, health-related quality of life, cardiovascular-related quality of life, physical activity

## Abstract

Background: Breast cancer (BC) and cardiovascular (CV) disease share many risk factors associated with worse outcomes, in terms of cancer relapse, CV events, and quality of life (QoL), that could be counteracted by physical exercise (PE). We aimed to assess the impact of a 12-week differential PE protocol on cardiometabolic profile, QoL, CV- and BC-related long-term outcomes, and physical activity (PA) in a cohort of BC survivors (BCS) not treated with chemotherapy. Methods: 57 BCS participated in a 12-week PE protocol [aerobic exercise training (AET) or resistance exercise training (RET)]. Anthropometric and CV evaluation, health-related (HR)-QoL, daily PA, cortisol, and dehydroepiandrosterone sulfate (DHEA-S) levels were assessed before (T0) and after (T1) PE. We assessed BC and CV outcomes, HR-QoL, CV-QoL, and PA at the follow-up. Results: RET improved waist circumference, DHEA-S, cortisol/DHEA-S, systolic and mean blood pressure, and ventricular/arterial coupling; AET ameliorated sagittal abdomen diameter and pulse wave velocity. Regarding HR-QoL, physical function improved only in AET group. At a mean 34 ± 3.6-month follow-up, we documented no significant differences in CV-QoL, HR-QoL, and PA or CV and BC outcomes. Conclusions: AET and RET determine specific, positive adaptations on many parameters strongly related to CV risk, CV and BC outcomes, and QoL, and should be included in any cardio-oncology rehabilitation program.

## 1. Introduction

Breast cancer (BC) is the most common malignancy and the leading cause of cancer-related death among women, although cardiovascular disease (CVD) still represents the most important source of mortality in the female population [1,2].

CVD and BC share several overlapping risk factors, such as age, tobacco habit, obesity, and sedentary behavior (SB), and CV health can influence both cancer outcomes and cancer treatment selection. Moreover, as long-term survival rates after diagnoses of BC are rising, physicians must face up to the long-term sequelae of the various therapeutic options for BC, that is, early or delayed cancer-therapy-related cardiovascular toxicity (CTR-CVT), potentially altering cancer survivorship. There are many clinical and subclinical manifestations of CTR-CVT; in particular, venous thrombosis, thromboembolism, peripheral atherosclerosis, dysrhythmia, valvular dysfunction, pericarditis, and heart failure have been described as complications of hormone therapy (HT) [3,4,5]. Adjuvant HT with aromatase inhibitors (AIs) or selective estrogen receptor modulators (SERMs, usually tamoxifen) exerts oncologic benefits by inhibiting estradiol synthesis or breast estrogen receptor signaling. AIs cause systemic estradiol depletion, whereas tamoxifen has mixed agonistic/antagonistic effects in a tissue-dependent fashion [6]. Since estrogens play a protective role in endothelial function, vascular tone, and cardiac function, as well as in lipid profile and inflammatory status, it looks intuitive that ET may modulate cardiometabolic risk and oxidative stress levels [7]. However, evidence about ET–associated cardiometabolic risks remains incomplete for many reasons. First, no randomized clinical trial has been designed to assess CV risk in this population. Second, clinical trials have described a low number of CV events and predominantly enrolled women with relatively low CV risk and with a short-term follow-up [6].

Apart from ET, cancer is associated with chronic inflammation, which is exacerbated by the effects of CTR-CVT, pre-existing CVD, and negative lifestyle risk factors, such as physical inactivity (PI). This persistent inflammatory status leads to endothelial dysfunction (ED), known as an early risk factor for atherosclerosis and arterial stiffness (AS) [8]. Physical exercise (PE) has positive effects on every aspect of BC progression, including all-cause and breast-cancer-related death. It represents a crucial tool to counteract the proinflammatory cancer-associated burden, to favorably influence the acute and chronic symptoms of BC and to ameliorate or prevent the development of cardiovascular (CV) risk factors and CVD as well [9,10,11,12]. Additionally, aerobic and resistance PE performed at moderate-to-high intensity can significantly improve health-related quality of life (HR-QoL) in breast cancer survivors (BCS) [13,14]. On the contrary, SB and PI have been associated with worsening HR-QoL in this population [15].

To date, many different protocols for physical activity (PA) and PE in oncologic patients have been proposed; however, the literature remains insufficient for further detailing prescriptions according to cancer type, types of treatment, and timing of treatment, and no conclusive data about the therapeutic efficacy of each exercise protocol are still available. Finally, there need to be more studies regarding the long-term effects of PE on both BC-related and CV-related outcomes, as well as on QoL and PA, for BCS patients treated with HT [9,16,17,18,19,20,21,22,23,24,25].

Therefore, our study aims to examine the effects of different PE protocols on cardio-metabolic profile, PA and QoL in a population of BCS, not treated with CHT; moreover, in the same population, we evaluated BC-related and CV-related outcomes, CV- and HR-QoL, and PA at a 34-month follow-up.

## 2. Materials and Methods

This is a single-center, prospective study in which, between April 2016 and April 2017, we screened 76 BCS patients at the Breast Cancer Surgery Unit of the ‘G. Bernabeo’ Hospital (Ortona, Italy). The inclusion criteria were age range between 50 and 65 years; history of BC surgery in the previous 12 months; no history of CHT; no ongoing radiotherapy (RT); eventual ET; lymphedema lower than class 2 of CEAP-L classification [26]; CV and orthopedic eligibility; no dieting or use of nutritional supplements; no participation in any exercise program during the six months prior to the study. The exclusion criteria were previous or ongoing CHT; any history of active CVD (i.e., ischemic heart disease, valvular heart disease, arrhythmias) or recent hospital admission for CVD; any systemic inflammatory disease or any orthopedic condition potentially limiting the physical training; abnormal exercise electrocardiography (ECG) stress test at the screening; dieting or use of nutritional supplements; participation in any exercise program during the six months prior to the study; nonemployed status. A Cardiologist and a Sports Medicine Specialist confirmed CV and orthopedic eligibility through a complete medical examination, 2-D transthoracic echocardiography (TTE) and maximal exercise ECG stress test. According to these criteria, 57 patients were considered eligible. The Ethics Committee of the ‘G. d’Annunzio’ University of Chieti-Pescara approved this study (#312/2015). In acquiescence with the Declaration of Helsinki, all enrolled patients gave written informed consent at the time of their evaluation, stating that data and images may be subsequently used for research purposes (Clinicaltrials.gov registration number: NCT04337736). 

Each eligible patient underwent an anthropometric assessment, complete CV evaluation, health-related quality of life (HR-QoL) analysis, a five-day PA recording in a free-living context, and salivary samples collection before (T0) and after (T1) physical training.

**Figure 1:**
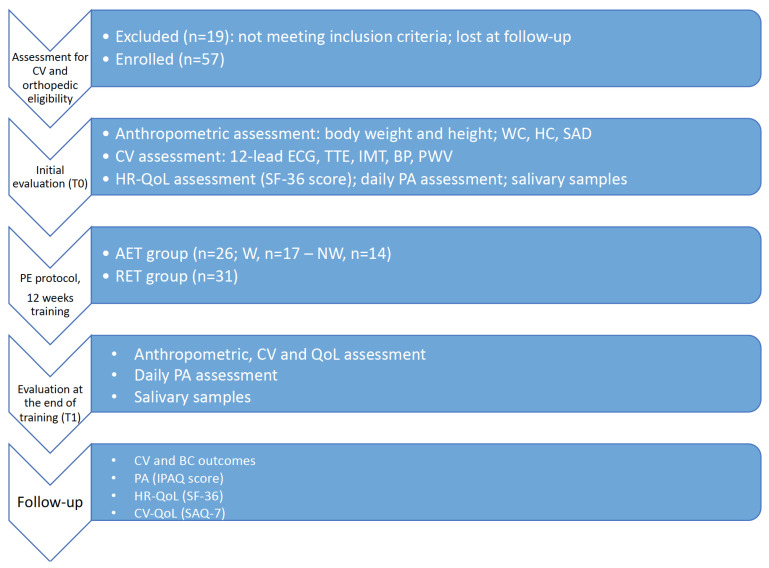
Flowchart of the study protocol. Abbreviations: CV, cardiovascular; WC, waist circumference; HC, hip circumference; SAD, sagittal abdomen diameter; ECG, electrocardiogram; TTE, transthoracic echocardiogram; IMT, intima-media thickness; BP, blood pressure; PWV, pulse wave velocity; AET, aerobic exercise training; W, walking; NW, Nordic walking; RET, resistance exercise training; QoL, quality of life; PE, physical exercise; PA, physical activity; IPAQ, International Physical Activity Questionnaire; SAQ-7, Seattle Angina Questionnaire-7; SF-36, Short Form Health Survey-36.

After a mean follow-up of 34 months, we evaluated CV and BC outcomes, PA, HR-QoL, and CV quality of life (CV-QoL) with a telephone interview (Figure 1). 

### 2.1. Anthropometric Assessment 

A second-level anthropometrist assessed bodyweight, stretched height, waist circumference (WC), and hip circumference (HC) according to the International Society for the Advancement of Kinanthropometry’s guidelines [27].

Body weight and stretched stature were measured to the nearest 0.1 kg and 0.1 cm, respectively, with the participants dressed in light clothing and without shoes and in fasting condition, using a stadiometer with a balance-beam scale (Seca 220; Seca, Hamburg, Germany). Body mass index (BMI) was calculated according to the formula by Du Bois et al. [28]. WC and HC were assessed with an anthropometric tape (Seca 200). WC was measured as the smallest circumference between the rib cage and the iliac crest at the end of normal expiration; in contrast, HC was measured at the level of the broadest circumference between the waist and the thighs [27]. Sagittal abdomen diameter (SAD) was measured with the subject lying supine, at the midpoint between the iliac crest and the last rib with a portable, sliding-beam abdominal caliper [29].

### 2.2. CV Eligibility and Complete CV Evaluation

CV eligibility was assessed through a 12-lead rest electrocardiogram, maximal exercise test, and TTE. 

After 10 min of supine rest, a 12-lead electrocardiogram (Stress ECG HD+, Cardioline, Trento, Italy) was achieved. 

Then, upon the supervision of a Sports Medicine specialist, participants performed a graded maximal exercise test, i.e., Astrand protocol using 3-min steps, on a cycle ergometer (Cardioline xr50, Cardioline, Trento, Italy), with continuous electrocardiogram monitoring (Stress ECG HD+, Cardioline, Trento, Italy) and blood pressure assessment at the end of each step. According to the Italian Federation of Sports Medicine guidelines, the test lasted until the doctor showed absolute or relative indications for clinically graded exercise test termination [30].

All the TTE were performed using a dedicated MyLab™30Gold CV portable ultrasound (Esaote, Florence, Italy) with a phased array 3.5 MHz cardiac probe. For each participant, general echocardiographic characteristics regarding cardiac chambers volumes and function, including left ventricular (LV) diastolic function and LV global longitudinal strain analysis (GLS), according to the recommendations from the American Society of Echocardiography and the European Association of Cardiovascular Imaging (EACVI) guidelines, were collected [31]. An offline and dedicated software (XStrain™ 2D) was used to perform the two-dimensional speckle-tracking analysis from the apical 4-chambers, 3-chambers, and 2-chambers view. Moreover, we assessed epicardial fat thickness as a marker of visceral adiposity and ventricular/arterial coupling (VAC) as an index of CV efficiency using the single beat method [32,33].

Carotid ultrasound (CUS) was performed using a standard 7.5 MHz linear probe, with each participant lying supine and the neck hyperextended. After manual image acquisition of carotid vessels, we assessed left and right intima-media thickness (IMT) on the common carotid posterior wall, 1 cm from bulb bifurcation on each side. Then, the Quality Intima Media Thickness (QIMT^TM^) software was used for data analysis, and the mean value between the three measurements for QIMT was obtained for each patient, with a concordance correlation coefficient between the three measurements of 0.99 (95% CI: 0.98–0.99) [34,35,36,37].

Pulse wave velocity (PWV), an index of AS, was evaluated with the photoplethysmographic method (Vascular Explorer, Enverdis, Dusseldorf, Germany), along with systolic, mean, and diastolic blood pressure (SBP, MBP, DBP) with the subject supine for at least 10 min before starting the assessment in a quiet, temperature-controlled room [34,38,39].

All the participants have been examined utilizing TTE, CUS, and photoplethysmographic method before and after the PE protocol (T0-T1). 

### 2.3. Quality-of-Life Assessment

The Italian version of the Short Form Health Survey (SF-36) score was used for the assessment of HR-QoL at T0 and T1. This 36-item patient-reported survey offers a consistent and acceptable description of general health status in the general population and in BCS [40,41]. It comprises eight scaled scores (PF, physical function; SC, social function; MH, mental health; P, pain; RLP, role limitation physical; RLM, role limitation mental; EV, vital energy; HP, health perception), which are the weighted sums of the questions in their section. Each scale is directly transformed into a 0–100 score, with a score of 0 equivalent to a maximum disability and a score of 100 equivalent to no disability. 

### 2.4. Daily Physical Activity Measurements

Daily PA was determined under free-living circumstances over five consecutive days, including four weekdays and one weekend day, by using SenseWear Pro3 armbands (BodyMedia, Pittsburgh, PA, USA). This device incorporates the information collected by the two-axis accelerometers and sensors (i.e., skin and near-body temperature, heat flux, and galvanic skin response) with the sex, age, stature, weight, smoking status, and handedness of the user. Thus, it offers details about the intensity and the amount of the daily PA. From the recorded data, we focused on the mean intensity of daily PA (METs), daily steps (Steps), time spent on PA with intensity from 3 to 6 METs (i.e., moderate-intensity PA; MIPAT), and time spent on PA with intensity from 6 METs to 9 METs (i.e., vigorous-intensity PA; VIPAT). In addition, the time spent in sedentary and low-intensity PA (LIPAT) was also considered. The time spent in PA with an intensity < 1.5 METs, excluding nocturnal sleeping, was considered sedentary time (ST), whereas the time spent in physical activities >1.5 METs and <3 METs was considered LIPAT [42]. The participants wore their monitors all day, except while bathing. No rainy days occurred during the recorded periods. The wear time criteria for valid registrations were at least 540 min/day on weekdays and 480 min/day on weekends [43].

### 2.5. Salivary Samples

Salivary samples were collected three times under a free-living setting using a Salimetrics oral swab (Salimetrics Europe, Suffolk, UK), i.e., a small pad, absorbent and nontoxic, passed within the oral cavity for 2–3 min before being placed inside a labeled tube. The samples were stored at 8 a.m., 5 p.m., and 11 p.m.; refrigerated within 30 min; and frozen at −20 °C within 2 h of collection. On the day of the assay, samples were thawed and centrifuged for 15 min at 3000 rpm to extract saliva and remove the mucin, and the swab was then discarded. Each sample was processed in duplicate. Salivary samplings from 8 a.m. were collected in fasting condition, except for water consumption; in contrast, those from 5 p.m. and 11 p.m. were collected 2 h after any food or beverage consumption, except for water. The sum of the three samplings results was considered representative variable data of daily salivary cortisol excretion [44]. The assays were performed using the High Sensitivity Salivary Cortisol EIA kit and Salivary DHEA-S EIA kit (Salimetrics Eu-rope, Suffolk, UK), with an intra-assay coefficient of variation of 4.6% for cortisol and 7.25% for DHEA-S and an interassay precision of 6% for cortisol and 7.5% for DHEA-S.

### 2.6. Follow-Up Evaluation

At the follow-up, during a telephone interview, we evaluated HR-QoL, CV and cancer outcomes, CV-QoL, and PA. The Italian version of the Short Form Health Survey (SF-36) score was used to evaluate HR-QoL [40,41]. CV and cancer outcomes were assessed with questions that included hospitalization for CV issues, initiation of novel CV medications, and BC recurrency. CV-QoL was estimated with telephone interview through Seattle Angina Questionnaire-7 (SAQ-7), a shortened version of the full SAQ, previously validated both in the male and female population. This questionnaire includes seven questions to quantify health status in patients with coronary artery disease (CAD). It focuses on three domains that directly evaluate patients’ current health status: Physical Limitation (PL), Angina Frequency (AF), and Quality of Life (QL). Each domain results in a score (from 0 to 100, with 0 denoting the worst and 100 the best possible health status) with an overall final summary score [45,46,47].

Last, International Physical Activity Questionnaire—Short Form (IPAQ-SF), whose reliability and validity have been previously recognized, was used to measure physical activity (PA) at the follow-up. It consists of seven questions to estimate the average daily time spent sitting, walking, and engaging in moderate and vigorous PA over the last seven days. Activities that require up to 3 Metabolic Equivalent of Task (METs) have been defined as light-intensity PAs, and activities that range from 3 to 6 METs have been categorized as moderate-intensity PAs; in contrast, those that require more than 6 METs were defined as vigorous-intensity PAs. The IPAQ-SF sum score is then expressed in PA MET/minutes per week [48,49,50,51].

### 2.7. Physical Exercise Protocols

After basal evaluation and eligibility, BCS were allocated to one of two aerobic exercise protocols (AET), i.e., walking (W) or Nordic walking (NW), or resistance exercise training (RET). We assigned every participant to each PE protocol according to the season, i.e., the women enrolled during spring or summer were assigned to the AET and trained outside, whereas the women enrolled during autumn or winter were assigned to the RET and trained inside in a dedicated gym. 

Each group was conducted and supervised by exercise specialists. Total adherence (TA) to PE protocol was expressed as the percentage of total exercise volume (ExV) performed on planned total ExV. 

#### 2.7.1. Walking (W) Group 

Patients assigned to W group worked out at moderate intensity for 12 weeks, 4-days a week. Exercise intensity was distributed and supervised, as stated in the ratings of the perceived exertion method [52]. From the first to the fourth week, each training session lasted 40 min, with a walking velocity eliciting an effort equal to 10–11 according to the 15-category rating of the perceived exertion scale (RPE). Then, the participants trained for 50 min per each training session at 12–13 RPE from the fifth to the eighth week; from the ninth to the twelfth week, only the training intensity improved from 12–13 to 13–14 RPE. The participants were familiarized with this scale before beginning the training and during the first week of training as well. The Borg scale is particularly helpful to prescribe and monitor exercise intensity in this population because for the same external load, it is possible to have a different internal load, due, for example, to side effects of pharmacological treatments (i.e., fatigue). Exercise trainers checked the exercise intensities of the participants through the talk test and tested their compliance with the training sessions [53].

#### 2.7.2. Nordic Walking (NW) Group 

NW participants were introduced to the protocol through 10 lessons on the NW technique, before starting the 12 weeks of supervised workouts. The central part of each lesson, lasting 35 min, shifted its main content from the practice of exercises to the practice of the complete NW technique, maintaining its intensity lower than 10, according to Borg’s rating of perceived exertion scale (RPE) [52]. At the end of this introductory course, the technique appropriateness of each participant was independently verified by two NW instructors of the International Nordic Walking Association (INWA). Sub-groups followed the same training scheme with different contents according to sub-group membership: 12 weeks of supervised training, with a three-times per week cadence; 70 min of training including 15 min of warm-up, 45 min of central phase, and 10 min of cool down. Participants trained from the first to the fourth week at 10–11 RPE, from the fifth to the eighth week at 12–13 RPE, and from the ninth to the twelfth week at 13–14 RPE. The participants were familiarized with Borg’s scale before beginning the training and during the first week of training as well. Compliance with the training sessions was tested through both women’s and exercise trainers’ diaries. 

#### 2.7.3. Resistance Exercise Training (RET) Group

RET protocol was structured in 28 sessions, each lasting 50 min. The first six lessons were focused on postural and respiratory training; then, from the 7th to the 16th lessons, each workout was organized in a circuit training manner including eight different exercises, involving the major muscle groups, and executed in a calisthenics way or using the elastic bands. Each exercise lasted 50 s, a rest period of 2 min was observed from one exercise to another. From the 17th to the 28th lesson, just the duration of each exercise (1 min) and that of the rest period (1 min) have been modified. Participants trained from the first to the fourth week at 10–11 RPE, from the fifth to the eighth week at 12–13 RPE, and from the ninth to the twelfth week at 13–14 RPE. The participants were familiarized with Borg’s scale before beginning the training and during the first week of training as well. Compliance with the training sessions was checked through both women’s and exercise trainers’ diaries.

### 2.8. Statistical Analysis

Continuous variables are shown as mean and standard deviation (SD) or median and lower/upper limit (Q1, Q3), while categorical data as absolute numbers and percentages, as appropriate. Adjusted means and standard errors (SE) resulted from linear/logistic regression models. All the variables were tested for normality using the Shapiro–Wilk test. We distributed the participants into two groups according to PE protocol: AET, aerobic exercise training; RET, resistance exercise training. Then, we estimated the absolute change for each variable using the following formula: postexercise program value minus the pre-exercise program value divided by the pre-exercise program, to normalize for T0 values all the variables. According to their distribution, differences between groups and absolute changes were assessed utilizing the Student’s *t*-test or a nonparametric *t*-test. A two-tailed *p*-value of 0.05 was considered statistically significant. 

All the statistical analyses were adjusted by age, hormone therapy (HT), radiotherapy (RT), BMI, body surface area (BSA), TA, and ST, to exclude any confounding factors potentially influencing our results. Statistical analysis was completed using the SPSS software package (SPSS 22.0, Chicago, IL, USA) and Prism 6.0 (GraphPad Software, La Jolla, CA, USA). 

## 3. Results

Table 1 shows the baseline characteristics of our population. 

TA to PE protocol was 72.14 ± 24.5%, with a higher TA in RET group as compared to AET group (79.3 ± 13.8% vs. 66 ± 29.9%, *p* = 0.042). 

Table 2 shows the cardiometabolic profile, daily PA levels, and the QoL scores of our population, as for PE protocol (AET or RET), before (T0) and after training (T1). 

RET group experienced a statistically significant improvement in WC (*p* < 0.001), DHEA-S (*p* = 0.002), cortisol (*p* = 0.002), cortisol/DHEAS (*p* < 0.001), IMT (*p* = 0.005), and epicardial fat (*p* = 0.003), whereas AET group ameliorated DHEAS (*p* = 0.002) and epicardial fat (*p* < 0.001). 

Among CV parameters, in the RET group, we found an improvement in SBP (*p* = 0.004), DBP (*p* = 0.003), MBP (*p* = 0.001), RWT (*p* = 0.02), MAPSE (*p* = 0.003), GLS (*p* = 0.01), LAEF (*p* = 0.005), SV (*p* = 0.002), EA (*p* = 0.006), EES (*p* < 0.001), V/A (*p* < 0.001); AET group showed an improvement in PWV (*p* < 0.001), GLS (*p* < 0.001), and SV (*p* = 0.006).

Regarding daily PA levels, AET group improved METs (*p* = 0.008) and VIPAT (*p* = 0.04); in contrast, RET group ameliorates STEPs (*p* = 0.004). No significant differences where demonstrated in term of ST in both groups. 

QoL scores demonstrated an improvement in PF (*p* < 0.001), P (*p* = 0.001), HP (*p* > 0.001) in AET group and in SC (*p* = 0.03) and MH (*p* = 0.003) in RET group.

At the analysis of delta absolute change, adjusted for age, BSA, BMI, HT, RT, ST, and TA (Table 3), RET group showed a significant reduction of WC (*p* = 0.014), DHEAS (*p* = 0.013), and cortisol/DHEAS (*p* = 0.001), whereas AET group showed a significant reduction in SAD (*p* = 0.019). Among CV parameters, RET group showed an improvement in SBP (*p* = 0.021), MBP (*p* = 0.038), EES (*p* = 0.001), EA (*p* = 0.026), and V/A (*p* = 0.001), whereas AET group showed an improvement in PWV (*p* = 0.015). Finally, among QoL score, only AET group showed an improvement in PF (*p* = 0.03).

After dividing the population as for PE subgroups (Table 4), at the analysis of delta absolute change, we found that only RET group experienced a significant improvement in WC (*p* = 0.004), DHEAS (*p* = 0.026), cortisol/DHEAS (*p* = 0.001), SBP (*p* = 0.017), EES (*p* = 0.001), and V/A (*p* = 0.001), whereas NW group improved PWV (*p* = 0.005).

Table 5 shows the follow-up data in general population and in PE subgroups (AET and RET); 21 patients were lost at the follow-up. The mean follow-up was 34 ± 3.6 months in the whole population. Regarding BC outcomes, two BC recurrences were described, one in the AET group and one in the RET group; in contrast, two patients required the introduction of novel CV medications, and one patient was hospitalized for CV issues, both in the AET group. No significant differences in SAQ-7 score, HR-QoL, and PA measured in MET-minutes per week were found between the two groups at the follow-up. The mean IPAQ-score was 2160 in the whole population, indicative of a sufficiently active lifestyle. Participants in the RET group experienced a higher percentage of low-level PA (*p* = 0.012). 

## 4. Discussion

To the best of our knowledge, our study is the first explorative analysis focused on the effects of a 12-week supervised PE protocol on cardiometabolic status, QoL, and PA in a population of BCS, not treated with anthracyclines or HER-2 inhibitors, providing data on BC and CV outcomes, PA, and QoL at a 34-month follow-up.

According to our results, a 12-week supervised PE protocol was associated with an improvement in cardiometabolic profile, with peculiar differences according to the type of PE. In particular, RET determined with an improvement in WC, DHEA-S, cortisol/DHEA-S, SBP, MBP, and V/A coupling; in contrast, AET ameliorated SAD and PWV. Among HR-QoL parameters, we only observed an improvement of PF in the AET group. At the follow-up analysis, no differences were documented in CV-QoL, HR-QoL, and PA, according to PE subgroups.

Central or abdominal obesity, measured as WC or SAD, represents one of the risk factors for diagnosing metabolic syndrome and is strongly related to mortality and cardiorespiratory fitness [54,55,56,57]. Pischon et al. demonstrated, in a group of 359,387 participants from the European Prospective Investigation into Cancer and Nutrition (EPIC) population, that a 5-cm increase of WC leads to an increased higher risk of death of 17% for men and 13% for women [58]. Moreover, Dyrstad et al. highlighted that even a small increase in WC is associated with a significant reduction in cardiorespiratory fitness, the latter being a meaningful marker of CV health in BCS [59,60]. SAD has been identified as a robust anthropometric measure of visceral adipose fat, being considerably associated with insulin resistance and cardiometabolic risk [61,62]. Additionally, in the premenopausal women population, SAD correlates with epicardial adipose tissue [63]. About 65% of BCS are overweight or obese, with a significant risk for cancer relapse and all-cause mortality. Furthermore, both medical anticancer therapy and the inactivity related to cancer could increase the distribution of fat mass, with a reduction in lean mass and bone density. On the other hand, PE could counteract any increase in WC and SAD, as demonstrated in our study, in line with the available data in the literature [64,65,66].

The effects of PE on the hormonal milieu in healthy women have been widely demonstrated, with a decrease in circulating sex hormones potentially protective from the risk of breast cancer [67,68]. In cancer patients, PE may correlate to endogenous stress reduction, which is related to many cardiometabolic outcomes and psychophysical health [69,70]. Many studies demonstrated that AET reduces cortisol level in BCS, whereas there are still insufficient data about the effects of different PE protocol on DHEA-S level in this population [71,72,73]. Our results demonstrated that a RET protocol could significantly reduce DHEA-S and cortisol/DHEA-S levels, regardless of HT, with a possible protective role on cancer relapse, as androgen receptors modulate BC cell growth both with a genomic and nongenomic pathway [74]. However, these results should be confirmed and analyzed at a long-term follow-up, as a potential anti-metastatic role for DHEA-S in BC has been recently advocated [75].

Among CV parameters, much evidence documented the role of RET in the reduction of SBP, both in hypertensive patients, postmenopausal women with and without arterial hypertension, and BCS, with a strong recommendation from the European Society of Cardiology (ESC) to encourage the incorporation of RET both in primary and secondary CV prevention PE protocol [34,76,77,78,79]. This effect could be mediated by peripheral vascular resistance and arterial stiffness reduction, renal and muscular sympathetic activity decline, and modulation of cardiac and renal baroreceptors [80]. Our data align with the available evidence in the literature [81,82]. VAC, as the ratio between end-systolic ventricular elastance (Ees) and arterial elastance (Ea), presents a marker of CV efficiency, with an independent diagnostic and prognostic value in CVD and CV risk stratification [83]. VAC reduces with increasing age and in the presence of CV risk factors, such as increased aortic rigidity or left ventricular diastolic dysfunction, especially in the female population [84,85]. PE can reverse these negative adaptations, in a dose-dependent manner, even in the older population, with a potentially more significant effect on women [86,87,88]. Moreover, a differential effect of AET and RET on VAC has been advocated, with AET being associated with increased brachial artery absolute diameter and blood flow during hyperemia and RET inducing changes in vascular reactivity [89].

Besides CV risk factors, even CHT can worsen VAC in BCS, with a reduction in both Ees and left ventricular systolic function [90,91,92]. However, to date, there is no evidence about the effects of PE on VAC in BCS. Our data provide promising results about RT’s role in improving VAC, both in terms of Ea and Ees, in BCS not treated with CHT.

PWV represents the gold standard for assessing arterial stiffness (AS) and is inversely correlated with vascular compliance [93]. AS represents one of the earlier markers of structural and functional vascular degeneration and is associated with a worsening of CV outcomes, regardless of traditional CV risk factors assessed by Framingham Risk Score [94]. The female population experiences an earlier and more severe increase in AS according to age than the male population, with a two-fold higher risk of mortality in women than men [95]. As for VAC, PE can reverse PWV worsening associated with age and CV risk factors [96]. Among PE protocols, AET has been associated with a significant improvement in PWV, for its role in the endothelium-derived nitric oxide function, both in the general population and postmenopausal women [97,98,99]. In the specific setting of BCS, both CHT and RET can determine a significant worsening of PWV, especially in patients treated with aromatase inhibitors [100,101]. To the best of our knowledge, the role of PE on PWV in BCS treated with HT still needs to be clarified. Our data show a potentially relevant role of AET in PWV improvement, regardless of HT.

Several studies have recognized the benefits of PE in HR-QoL in BCS [102]. In particular, a longer exercise session duration, a combination of PE protocol and a supervised intervention could determine the most significant improvement in HR-QoL [103,104,105]. However, according to a meta-analysis by Lahart et al., PE can determine only a small-to-moderate significant improvement in HR-QoL that does not persist longer than 12 weeks [106]. In our cohort, we found a significant improvement in physical function after PE training, but only in the AET subgroup. Moreover, at the follow-up, we found no differences between the PE subgroups regarding CV-QoL and HR-QoL, in line with the data available in the literature. Regarding PA, we observed an increase in METs and VIPAT in AET group and an increase in STEPS/day in RET group; in contrast, we did not document any compensatory increase in ST after PE training. Regarding follow-up data, the IPAQ-score indicated a sufficiently active lifestyle in both the general population and PE subgroups, with a higher percentage of inactivity in RET group. These data underline the need for a call to action to increase motivation for PA in BCS, as many research groups already addressed [107].

The limitations of our study were: the small sample size; the assessment of epicardial fat and sagittal abdominal diameter, respectively, with transthoracic echocardiography and anthropometric evaluation instead of computed tomography; and the evaluation of spontaneous PA with IPAQ score at the follow-up instead of accelerometry.

The latest guidelines on cardio-oncology, recently published by the ESC, strengthened the noteworthy interconnection between CVD and cancer in terms of common modifiable and nonmodifiable risk factors, as well as regarding the importance of maintaining a healthy lifestyle and adequate cardiorespiratory fitness (CRF) after cancer diagnosis and during and after anticancer therapy. In particular, the guidelines recommend maintaining adequate PA and underline that exercise prescription represents a potent multitargeted tool capable of counteracting anticancer treatment side effects, with a potential impact both in primary as well as secondary prevention of CTR-CVT and a paramount role in primary and secondary prevention of CV risk factors [8].

Moreover, the guidelines emphasize that, to date, current evidence does not support a specific protocol of PE, as exercise prescription should be tailored to the specific patient’s fitness level and basal characteristics and systematically improved to enhance physiological adaptation to get the best results both in terms of CRF, prevention, and treatment of anticancer side effects and prevention of cancer relapse as well [25,108,109].

Our data showed, for the first time in the literature, the effects of different PE protocols on cardio-metabolic parameters, QoL, and PA in a BCS population not treated with CHT or HER-2 inhibitors, with data about CV and BC outcomes, PA, CV-QoL, and HR-QoL at a 34-month follow-up. AET and RET can determine specific, positive adaptations on many parameters strongly related to CV risk and outcomes and should be included in every cardio-oncology rehabilitation program. Ideally, each BCS should be involved in a dedicated cardio-oncology rehabilitation program tailored to the specific patient’s cardio-metabolic status, including intense, motivational counseling, to get the best compliance with exercise prescription, maintain an adequate PA level, and achieve the most remarkable effects on both short and long-term BC and CV outcomes and QoL [110].

## Figures and Tables

**Table 1:** General characteristics of the study population, according to PE protocol.

	General Population	AET	RET	*p*-Value
	(n = 57)	(n = 31)	(n = 26)	
Age, years	53.1 ± 6.8			
TA, %	72.14 ± 24.5	66.0 ± 29.9	79.3 ± 13.8	**0.042**
PE protocol, n (%)				**0.001**
AET	31 (54.4%)	14 (45.2%)	0 (0%)	
W	17 (29.8%)	0 (0%)	26 (100%)	
NW	14 (24.6%)	17 (54.8%)	0 (0%)	
RET	26 (45.6%)			
BC recurrence, n (%)	2 (3.6%)	2 (6.5%)	0 (0%)	0.19
RT, n (%)	19 (33.3%)	7 (22.6%)	12 (46.2%)	0.06
HT, n (%)	39 (68.4%)	17 (54.8%)	21 (80.8%)	**0.039**
AIs, n (%)	24 (42.2%)	8 (25.8%)	16 (61.5%)	**0.039**
Tamoxifen, n (%)	15 (27.3%)	10 (32%)	5 (19.2%)	0.27
CV therapy, n (%)				
Statins, n (%)	1 (1.7%)	1 (3.2%)	0 (0%)	0.36
BB, n (%)	6 (10.5%)	1 (3.2%)	5 (19.2%)	0.05
ACE-I, n (%)	11 (19.3%)	4 (12.9%)	7 (26.9%)	0.18
APT, n (%)	4 (1.7%)	3 (9.7%)	1 (3.9%)	0.39
CCB, n (%)	1 (1.7%)	1 (3.2%)	0 (0%)	0.36
Diuretics, n (%)	6 (10.5%)	2 (6.5%)	4 (15.4%)	0.27
SF-36				
PF	80 [65, 85]	80 [60, 85]	80 [70, 90]	0.22
SC	75 [62, 87]	75 [50, 100]	75 [62, 87]	0.25
MH	64 [52, 76]	64 [48, 76]	64 [52, 80]	0.45
P	52 [41, 74]	52 [41, 74]	52 [41, 84]	0.59
RLP	75 [25, 100]	50 [25, 100]	75 [10, 100]	0.95
RLM	66 [33, 100]	66 [0, 100]	66 [33, 100]	0.67
EV	55 [45, 65]	55 [40, 65]	55 [45, 65]	0.44
HP	56 [42, 72]	55 [42, 82]	56 [40, 72]	0.72

List of the abbreviations: AET, aerobic exercise training; RET, resistance exercise training; W, walking; NW, Nordic walking; TA, total adherence to PE program; RT, radiotherapy; HT, hormone therapy; AIs, aromatase inhibitors; CV, cardiovascular; BB, beta-blockers; ACE-I, ACE-inhibitors; APT, antiplatelet therapy; CCB, calcium channel blockers; SF-36, Short Form Health Survey-36; PF, physical function; SC, social function; MH, mental health; P, pain; RLP, role limitation physical; RLM, role limitation mental; EV, vital energy; HP, health perception. Bold values denote statistical significance at the *p* < 0.05 level.

**Table 2:** Pretraining (T0) and post-training (T1) cardiometabolic, QoL profile, and PA levels of the study population, according to PE protocol.

	AET			RET		
	n = 31			n = 26		
	T0	T1	*p*-Value	T0	T1	*p*-Value
Metabolic parameters						
Weight, kg	68 ± 11.7	67.3 ± 12.1	0.2	65.4 ± 13.4	66.1 ± 13.6	0.11
BMI	26.2 ± 5	-	-	25.2 ± 5.08	-	-
HC, cm	100.6 ± 20.9	99.5 ± 21.01	0.13	103.3 ± 9.5	101.4 ± 9	0.06
WC, cm	81.1 ± 18.3	80.3 ± 18.3	0.05	89.6 ± 11.2	82.4 ± 11.06	**<0.001**
SAD, cm	25.4 ± 6.2	25.1 ± 6.1	0.28	25.6 ± 3.5	25.4 ± 3.9	0.15
DHEAS, ug/dL	0.29 ± 0.22	0.20 ± 0.15	**0.002**	0.47 ± 0.6	0.33 ± 0.47	**0.002**
Cortisol, ug/dL	0.10 ± 0.06	0.10 ± 0.08	0.79	0.15 ± 0.1	0.07 ± 0.06	**0.002**
Cortisol/DHEAS	0.67 ± 1.1	0.57 ± 0.5	0.6	0.78 ± 0.8	0.001 ±0.006	**<0.001**
IMT, mm	0.67 ± 0.12	0.65 ± 0.08	0.05	0.69 ± 0.1	0.61 ± 0.14	**0.005**
Epicardial fat, mm	8.1 ± 2.6	6.5 ± 1.4	**<0.001**	8.4 ± 2.3	6.7 ± 1.3	**0.003**
CV parameters						
SBP, mmHg	130.3 ± 16.9	130 ± 13.8	0.9	132.6 ± 20.9	124 ± 17.8	**0.004**
DBP, mmHg	79.7 ± 11.2	78.9 ± 11.3	0.67	80 ± 12.8	74.3 ± 12.7	**0.003**
MBP, mmHg	96.6 ± 12.3	95.9 ± 11.5	0.9	97.5 ± 14.9	90.9 ± 13.8	**0.001**
PWV, m/sec	9.5 ± 2.9	6.9 ± 3.2	**<0.001**	8.6 ± 2.2	7.8 ± 2.2	0.19
HR, bpm	74.2 ± 10.2	71.7 ± 9.6	0.05	73.6 ± 9.2	71.1 ± 10.2	0.12
RWT	0.4 ± 0.04	0.38 ± 0.05	0.15	0.35 ± 0.06	0.37 ± 0.04	**0.02**
E/E’	7.6 ± 3.9	6.2 ± 2.7	0.3	6.6 ± 2.1	5.9 ± 1.8	0.3
TAPSE, mm	22.9 ± 3.9	23.7 ± 3.8	0.5	23.4 ± 3.3	23.1 ± 3.7	0.7
MAPSE, mm	14.9 ± 1.9	15.5 ± 2.2	0.33	15.7 ± 2.2	17.4 ± 2.6	**0.003**
GLS, %	22.2 ± 3.9	26.3 ± 3.3	**<0.001**	22.1 ± 2.6	25.7 ± 3.2	**0.01**
PAPs, mmHg	27.6 ± 5.4	28.5 ± 2.9	0.5	26.6 ± 2	24.4 ± 3.1	0.45
LA EF, mL	53.8 ± 9.2	55.2 ± 7.4	0.5	58.3 ± 9.05	66.7 ± 9.6	**0.005**
LVEF, %	60.2 ± 5.2	59.8 ± 4.8	0.9	64 ± 3.9	63 ± 4	0.4
FAC, %	0.45 ± 0.07	0.45 ± 0.08	0.7	0.47 ± 0.06	0.5 ± 0.06	0.18
SV, mL	48.9 ± 9.3	55.4 ± 10.1	**0.006**	51 ± 10.6	60 ± 12.5	**0.002**
EA	2.5 ± 0.7	2.2 ± 0.5	0.15	2.3 ± 0.5	1.8 ± 0.8	**0.006**
EES	2.3 ± 0.8	2 ± 0.6	0.18	1.93 ± 0.5	1.1 ± 0.3	**<0.001**
V/A	1.14 ± 0.3	1.2 ± 0.3	0.6	2.4 ± 0.9	1.2 ± 0.4	**<0.001**
SF-36						
PF	80 [60, 85]	85 [75, 95]	**<0.001**	80 [70, 90]	87.5 [80, 95]	0.05
SC	75 [50, 100]	75 [50, 100]	0.9	75 [62, 87]	87 [75, 100]	**0.03**
MH	64 [48, 76]	64 [52, 80]	0.05	64 [52, 80]	74 [68, 84]	**0.003**
P	52 [41, 74]	74 [52, 84]	**0.001**	52 [41, 84]	72 [61, 84]	0.26
RLP	50 [25, 100]	100 [0, 100]	0.1	75 [10, 100]	100 [54, 100]	0.13
RLM	66 [0, 100]	100 [66, 100]	0.06	66 [33, 100]	100 [66, 100]	0.13
EV	55 [40, 65]	60 [45, 70]	0.04	55 [45, 65]	55 [50, 75]	0.17
HP	55 [42, 82]	67 [52, 86]	**<0.001**	56 [40, 72]	67 [52, 76]	0.13
Daily PA assessment						
METs, avg	1.37 ± 0.23	1.46 ± 0.25	**0.008**	1.32 ± 0.17	1.38 ± 0.25	0.18
STEPS (no.)	10,003 [8084, 11,401]	10,057 [8431, 13,212]	0.8	8339 [7077, 9611]	10,348 [8185, 13,050]	**0.004**
LIPAT (min)	275 [154, 385]	334.5 [192, 402]	0.29	250 [171, 329]	290 [170, 426]	0.8
MIPAT (min)	45.5 [25, 87]	64 [37, 119]	0.07	47.5 [23, 81]	49.5 [39, 103]	0.23
VIPAT (min)	0 [0, 7]	2,5 [0, 13]	**0.04**	0 [0, 4]	0 [0, 8]	0.58
ST (min)	1036.5 [913, 1207]	977 [862, 1125]	0.05	1065 [1003, 1220]	1041 [974, 1199]	0.68

List of abbreviations: AET, aerobic exercise training; RET, resistance exercise training; BMI, body mass index; HC, hip circumference; WC, waist circumference; SAD, sagittal abdominal diameter; DHEA-S, dehydroepiandrosterone sulphate; IMT, intima-media thickness; SBP, systolic blood pressure; DBP, diastolic blood pressure; MBP, mean blood pressure; PWV, pulse wave velocity; HR, heart rate; RWT, relative wall thickness; TAPSE, tricuspid annular plane excursion; MAPSE, mitral annular plane excursion; GLS, global longitudinal strain; PAPs, systolic pulmonary artery pressure; LA EF, left atrium ejection fraction; LVEF, left ventricle ejection fraction; FAC, fractional area change; SV, stroke volume; EA, arterial elastance; EES, ventricular end-systolic elastance; V/A, ventricular/arterial coupling; SF-36, Short Form Health Survey-36; PF, physical function; SC, social function; MH, mental health; P, pain; RLP, role limitation physical; RLM, role limitation mental; EV, vital energy; HP, health perception; PA, physical activity; METs, metabolic equivalent of task; LIPAT, light intensity physical activity; MIPAT, moderate-to-intense. Bold values denote statistical significance at the *p* < 0.05 level.

**Table 3:** Delta absolute change of cardiometabolic and QoL parameters according to PE protocol.

	AET	RET	*p*-Value
	n = 31	n = 26	
Metabolic parameters			
WC, cm	(-)0.01 ± 0.01	(-)0.05 ± 0.02	**0.014**
SAD, cm	(-)0.01 ± 0.01	0.08 ± 0.03	**0.019**
DHEAS, ug/dL	0.05 ± 0.09	(-)0.32 ± 0.10	**0.013**
Cortisol/DHEAS	0.25 ± 0.12	(-)0.98 ± 0.13	**0.001**
CV parameters			
SBP, mmHg	0.001 ± 0.02	(-)0.06 ± 0.02	**0.021**
MBP, mmHg	(-)0.01 ± 0.02	(-)0.06 ± 0.02	**0.038**
PWV, cm/sec	(-)0.31 ± 0.08	(-)0.03 ± 0.08	**0.015**
EES	(-)0.04 ± 0.07	(-)0.40 ± 0.08	**0.001**
EA	(-)0.05 ± 0.05	(-)0.22 ± 0.06	**0.026**
V/A	0.12 ± 0.08	(-)0.41 ± 0.09	**0.001**
SF-36			
PF	0.2 ± 0.4	0.01 ± 0.1	**0.03**

List of the abbreviations: AET, aerobic exercise training; RET, resistance exercise training; WC, waist circumference; SAD, sagittal abdomen diameter; DHEA-S, dehydroepiandrosterone sulphate; CV, cardiovascular; SBP, systolic blood pressure; MBP, mean blood pressure; PWV, pulse wave velocity; EES, ventricular end-systolic elastance; EA, arterial elastance; V/A, ventricular/arterial coupling; SF-36, short form-36; PF, physical function. Bold values denote statistical significance at the *p* < 0.05 level.

**Table 4:** Delta absolute change of cardiometabolic and QoL parameters according to PE subgroups.

	NW	W	RET	*p*-Value
	n = 14	n = 17	n = 26	
Metabolic parameters				
WC, cm	(-)0.01 ± 0.01	(-)0.01 ± 0.01	(-)0.05 ± 0.02	**0.004**
DHEAS, ug/dL	0.14 ± 0.12	(-)0.10 ± 0.16	(-)0.30 ± 0.10	**0.026**
Cortisol/DHEAS	0.28 ± 0.16	0.19 ± 0.22	(-)0.9 ± 0.13	**0.001**
CV parameters				
SBP, mmHg	(-)0.03 ± 0.03	0.03 ± 0.02	(-)0.06 ± 0.02	**0.017**
PWV, cm/sec	(-)0.49 ± 0.11	(-)0.15 ± 0.10	(-)0.04 ± 0.07	**0.005**
EES	(-)0.15 ± 0.10	0.08 ± 0.10	(-)0.41 ± 0.07	**0.001**
EA	(-)0.09 ± 0.07	(-)0.01 ± 0.07	(-)0.23 ± 0.06	0.07
V/A	0.12 ± 0.12	0.12 ± 0.12	(-)0.41 ± 0.09	**0.001**

List of the abbreviations: NW, Nordic walking; W, walking; RET, resistance exercise training; WC, waist circumference; DHEA-S, dehydroepiandrosterone sulphate; CV, cardiovascular; SBP, systolic blood pressure; PWV, pulse wave velocity; EES, ventricular end-systolic elastance; EA, arterial elastance; V/A, ventricular/arterial coupling; SF-36, Short Form Health Survey-36; PF, physical function. Bold values denote statistical significance at the *p* < 0.05 level.

**Table 5:** Cardiovascular and cancer outcomes, physical activity, CV, and HR-QoL at the follow-up, according to PE protocol.

Follow-Up				
	General Population	AET	RET	*p*-Value
	n = 36	n = 16	n = 20	
Follow-up, months	34 ± 3.6	38.2 ± 1	31	**<0.001**
BC recurrency at FU, n (%)	2 (5.6%)	1 (6.2%)	1 (5%)	0.99
Hospitalization for CV issues, n (%)	1 (2.8%)	1 (6.2%)	0 (0%)	**<0.001**
Novel CV medications, n (%)	2 (5.6%)	2 (13.4%)	0 (0%)	**<0.001**
IPAQ	2160 [600–3990]	2160 [990.0, 3400.0]	2212.5 [540.0, 5730.0]	0.90
High level	15 (41.7%)	6 (37.5%)	11 (55%)	0.39
Low level	9 (25%)	2 (13.4%)	7 (35%)	**0.012**
Moderate level	11 (30.5%)	8 (50%)	1 (5%)	0.10
SAQ-7	100.0 [86.1, 100]	100 [100, 100]	88.9 [86.1, 100.0]	0.13
SAQ-PL	100.0 [58.3, 100]	101 [100, 100]	75 [58.3, 100]	0.26
SAQ-AF	100.0 [100, 100]	100 [100, 100]	100 [100, 100]	0.41
SAQ-QL	100.0 [100, 100]	100 [100, 100]	100 [100, 100]	0.41
SF-36				
PF	75 [65, 80]	75 [70, 85]	70 [65, 80]	0.56
SC	67 [44, 89]	56 [44, 100]	67 [56, 89]	0.64
MH	68 [52, 84]	72 [52, 84]	62 [52, 84]	0.51
P	67 [44, 89]	67 [44, 100]	61.5 [44, 89]	0.97
RLP	50 [25, 100]	75 [0, 100]	50 [25, 100]	0.88
RLM	67 [33, 100]	67 [33, 100]	67 [33, 100]	0.87
EV	50 [35, 70]	55 [35, 70]	47.5 [40, 75]	0.63
HP	55 [35, 80]	55 [40, 85]	50 [35, 80]	0.63

List of abbreviations: AET, aerobic exercise training; RET, resistance exercise training; BC, breast cancer; FU, follow-up; CV, cardiovascular; IPAQ, International Physical Activity Questionnaire; SAQ-7, Seattle Angina Questionnaire-7; SAQ-PL; SAQ-AF; SAQ-QL; SF-36, Short Form Health Survey-36; PF, physical function; SC, social function; MH, mental health; P, pain; RLP, role limitation physical; RLM, role limitation mental; EV, vital energy; HP, health perception. Bold values denote statistical significance at the *p* < 0.05 level.

## Data Availability

The data presented in this study are available on request from the corresponding author.
